# Sin Nombre Virus and Rodent Species Diversity: A Test of the Dilution and Amplification Hypotheses

**DOI:** 10.1371/journal.pone.0006467

**Published:** 2009-07-31

**Authors:** Christine A. Clay, Erin M. Lehmer, Stephen St. Jeor, M. Denise Dearing

**Affiliations:** 1 Department of Biology, Westminster College, Salt Lake City, Utah, United States of America; 2 Department of Biology, Fort Lewis College, Durango, Colorado, United States of America; 3 School of Medicine, University of Nevada Reno, Reno, Nevada, United States of America; 4 Department of Biology, University of Utah, Salt Lake City, Utah, United States of America; University of Liverpool, United Kingdom

## Abstract

**Background:**

Species diversity is proposed to greatly impact the prevalence of pathogens. Two predominant hypotheses, the “Dilution Effect” and the “Amplification Effect”, predict divergent outcomes with respect to the impact of species diversity. The Dilution Effect predicts that pathogen prevalence will be negatively correlated with increased species diversity, while the Amplification Effect predicts that pathogen prevalence will be positively correlated with diversity. For many host-pathogen systems, the relationship between diversity and pathogen prevalence has not be empirically examined.

**Methodology/Principal Findings:**

We tested the Dilution and Amplification Effect hypotheses by examining the prevalence of Sin Nombre virus (SNV) with respect to diversity of the nocturnal rodent community. SNV is directly transmitted primarily between deer mice (*Peromyscus maniculatus*). Using mark-recapture sampling in the Spring and Fall of 2003–2005, we measured SNV prevalence in deer mice at 16 landscape level sites (3.1 hectares each) that varied in rodent species diversity. We explored several mechanisms by which species diversity may affect SNV prevalence, including reduced host density, reduced host persistence, the presence of secondary reservoirs and community composition. We found a negative relationship between species diversity and SNV prevalence in deer mice, thereby supporting the Dilution Effect hypothesis. Deer mouse density and persistence were lower at sites with greater species diversity; however, only deer mouse persistence was positively correlated with SNV prevalence. Pinyon mice (*P. truei*) may serve as dilution agents, having a negative effect on prevalence, while kangaroo rats (*Dipodomys ordii*), may have a positive effect on the prevalence of SNV, perhaps through effects on deer mouse behavior.

**Conclusions/Significance:**

While previous studies on host-pathogen systems have found patterns of diversity consistent with either the Dilution or Amplification Effects, the mechanisms by which species diversity influences prevalence have not been investigated. Our study indicates that changes in host persistence, coupled with interspecific interactions, are important mechanisms through which diversity may influence patterns of pathogens. Our results reveal the complexity of rodent community interactions with respect to SNV dynamics.

## Introduction

Species diversity greatly impacts many ecosystem functions such as productivity, resistance to invasion, and stability [1]–[5]. Recently, it has been suggested that species diversity also plays a key role in governing pathogen prevalence. The two leading hypotheses (“Dilution Effect” and “Amplification” or “Rescue Effect”) are similar in that they suggest that changes in species diversity alter host-pathogen dynamics and, in turn, affect pathogen prevalence [6]–[8]. However, these hypotheses predict divergent outcomes. The Dilution Effect predicts that species diversity decreases pathogen prevalence through mechanisms such as decreased host density, reduced encounters between hosts, or reduced host survival [7]. In contrast, the Amplification Effect predicts increased pathogen prevalence with greater species diversity, through increased encounters between hosts [7], or through the presence of secondary hosts [7], [9]. Support for these models has been investigated primarily in vector-borne pathogens, such as *Borrelia burgdorferi* (the causative agent of Lyme disease) and Louping ill virus [6], [8]. Other investigations in plant-pathogen systems and directly transmitted wildlife diseases suggest that these phenomena may be widespread in pathogen dynamics [1], [10]–[13]. Despite the body of theoretical research outlining the patterns of the Dilution or Amplification Effect in host-pathogen systems, the fundamental mechanisms by which species diversity influences pathogen prevalence have not been thoroughly investigated.

We tested whether the prevalence of Sin Nombre virus (SNV), a hantavirus directly transmitted by its mammalian host, could be explained by either the Dilution or Amplification Effect. The primary reservoir for SNV is the deer mouse (*Peromyscus maniculatus*), though other *Peromyscus* species [14], [15] and the desert woodrat (*Neotoma lepida*)[16] potentially serve as secondary reservoirs. SNV transmission between rodents is primarily by direct contact, during aggressive behaviors such as biting and scratching, as evidenced by the strong correlation between SNV infection and scarring [17]–[20]. SNV infection in rodents is chronic, resulting in lifelong antibody production [21]. Rodents shed the virus in urine, saliva and feces [22], [23]. Human infection with SNV typically occurs through inhalation of aerosolized virus from rodent excrement [24], and can progress to hantavirus cardio-pulmonary syndrome (HCPS), a disease with 38% mortality [25]–[27].

The composition and relative abundance of species within a community can have profound effects on the behavior and density of deer mice, thereby altering the prevalence of SNV. For example, deer mice shift microhabitat use to avoid encounters with other species such as kangaroo rats (*Dipodomys* spp.), pinyon mice (*P. truei*) and pocket mice (*Perognathus parvus*) [28]–[33]. When encounters between deer mice and other species occur, they often result in fighting and other aggressive interactions [28]–[30], [32]. Long-term experiments manipulating species composition demonstrate that the presence of other species can greatly depress deer mouse density through competition for limited resources [34]–[36]. Such changes in deer mouse density are likely to affect the frequency and type of encounters between deer mice and with other rodent species in the community, which could then alter SNV prevalence. Furthermore, competition for limited resources in high diversity communities could result in reduced survival or increased dispersal of deer mice; such changes in the persistence of the primary SNV host at a site could, in turn, result in a reduction of SNV prevalence [7], [37].

We tested the Dilution Effect and Amplification Effect models by measuring SNV prevalence and rodent species diversity at several sites in Great Basin Desert, Utah. Previous studies suggest that species diversity reduces the prevalence of hantaviruses, including SNV [12], [13], [38], [39], although these studies did not identify the mechanisms driving such patterns. The juniper-sagebrush habitat of the Great Basin desert is particularly well suited for studies of this nature, as SNV prevalence (0–50%) spans the range seen across all habitat types [15], [17], [21], [40]–[42]. Within the Great Basin, rodent species diversity also varies considerably between sites within the same habitat type [43]. Low-diversity communities may consist of one or two species, including deer mice, while diverse communities have seven or more nocturnal rodent species of varying abundance. Secondary reservoirs such as pinyon mice (*P. truei*) and desert woodrats (*N. lepida*) co-occur with deer mice and may serve to “amplify” the prevalence of SNV [6]. However, the competence of these secondary reservoirs is currently unknown and is difficult to test given the Biosafety Level 4 facility required to work with animals infected with SNV. Conversely, heteromyid rodents such as Ord's kangaroo rat (*Dipodomys ordii*) and Western pocket mouse (*P. parvus*) also co-occur with deer mice; these rodents are not SNV reservoirs and can achieve relatively high population densities. Heteromyids are frequently present in high diversity communities and may “dilute” SNV prevalence.

We explored mechanisms such as changes in host density, altered host persistence (via survival or dispersal), as well as the presence of secondary reservoirs, by which species diversity may affect SNV prevalence in deer mice. We predicted that if SNV followed the Dilution Effect model, SNV prevalence would be highest in low diversity communities, possibly due to increased density or increased persistence of deer mice, the primary SNV host. Conversely, if SNV followed the Amplification Effect model, SNV prevalence would be highest in high diversity communities, possibly due to the presence of more secondary reservoirs or due to increased host encounters. Additionally, we examined the relationship between SNV prevalence and the abundance of non-deer mice to identify potential dilution or amplification agents, i.e., rodent species other than deer mice that may affect the dynamics of SNV.

## Results

At 16 sites, we captured 2,855 rodents of eight species over three years (Table 1). Gini-Simpson Index values ranged from 0.18 at a site comprised of deer mice and pocket mice, to 0.79 at a site consisting of seven rodent species (Table 1). During the study, SNV prevalence across sites ranged from 0.0% to 51.3% within a single season (Table 1). There was a negative relationship between species diversity and SNV prevalence in deer mice (LMM, estimate = −0.150, Z = −1.893, *P* = 0.05).

**Table 1:** Summary of species diversity, prevalence of Sin Nombre virus and density of deer mice (Peromyscus maniculatus) at 16 sites in central Utah sampled 2003–2005.

Site	Spring 2003			Fall 2003		
	Species Diversity (D)	SNV Prevalence	Deer Mouse Density	Species Diversity (D)	SNV Prevalence	Deer Mouse Density
TJ-3	.30	13.6	22.0	.18	9.1	27.5
TJ-4	.29	7.0	42.5	.22	5.9	25.5
LS-5	.73	18.5	13.5	.77	6.7	15.4
LS-6	.68	0.0	5.0	.64	0.0	3.0
LS-7	.46	17.7	24.41	.58	5.9	18.0
LS-8	.44	16.7	12.0	.56	10.0	10.0
LS-9	-	-	-	.67	9.1	11.0
LS-10	-	-	-	.66	7.7	13.0
LS-11	-	-	-	.68	11.1	4.5
UL-13	-	-	-	.54	0.0	10.0
UL-14	-	-	-	.77	0.0	3.0

Site	Spring 2004			Fall 2004		
	Species Diversity (D)	SNV Prevalence	Deer Mouse Density	Species Diversity (D)	SNV Prevalence	Deer Mouse Density
TJ-3	.20	29.3	17.5	.43	37.1	18.0
TJ-4	.20	16.3	24.5	.27	23.9	33.5
LS-5	.76	11.5	13.0	.73	4.8	12.0
LS-6	.66	0.0	4.0	-	-	-
LS-7	.70	25.9	13.5	.49	19.5	20.5
LS-8	.57	21.7	11.5	.56	29.2	12.0
LS-9	.56		5.0	.47	4.8	10.5
LS-10	.64	10.0	11.0	.77	15.0	10.0
LS-11	.63	0.0	1.5	-	-	-
UL-13	.66	15.4	3.8	-	-	-
UL-14	.69	30.4	4.5	-	-	-
TJ-15	-	-	-	.42	15.0	10.0
TJ-16	-	-	-	.43	33.3	7.5
TJ-17	-	-	-	.70	10.0	10.0
TJ-18	-	-	-	.74	13.3	7.5
TJ-19	-	-	-	.79	0.0	7.5

Site	Spring 2005			Fall 2005		
	Species Diversity (D)	SNV Prevalence	Deer MouseDensity	Species Diversity (D)	SNV Prevalence	Deer MouseDensity
TJ-3	.63	39.1	11.5	.67	11.5	12.5
TJ-4	.37	51.3	19.0	.59	19.5	21.0
LS-5	.70	10.3	14.5	.74	20.0	18.0
LS-6	-	-	-	-	-	-
LS-7	.42	14.3	17.5	.65	2.6	19.0
LS-8	.49	11.5	13.0	.57	15.0	20.0
LS-9	.65	0.0	4.4	.68	24.1	14.5
LS-10	.64	5.3	10.0	.61	25.0	4.4
TJ-15	.27	30.4	11.5	.36	9.7	30.5
TJ-16	.27	18.8	8.0	.20	7.0	28.0
TJ-17	.68	25.0	6.0	.54	25.0	16.0
TJ-18	.65	29.4	8.5	.46	9.2	32.5
TJ-19	.75	5.9	8.5	.73	7.4	13.5

Site identifier includes geographic location (TJ = Tintic Junction; LS = Little Sahara; UL = Utah Lake) and the numeric code (3–19). Species diversity was estimated using Simpson's Index (D) for each site per season. Prevalence of SNV was estimated as the number of deer mice positive for SN viral antibodies divided by the total number of deer mice sampled at each site per season x 100%. Density of deer mice (no./ha) was estimated using program DISTANCE. Dashes indicate sites that were not sampled in a particular season.

### Species diversity, deer mouse density and SNV prevalence

Deer mouse densities (estimated using DISTANCE) ranged from 1.5 to 42.5 mice/ha during the study (Table 1). We found a negative relationship between deer mouse density and species diversity (LMM, estimate = −28.50, Z = −5.28, *P*<0.01). We did not find a significant relationship between deer mouse density and SNV prevalence (LMM, estimate = 0.001, Z = 0.33, *P* = 0.74).

### Species diversity, deer mouse persistence and SNV prevalence

The proportion of deer mice that survived across seasons ranged from 0% to 27.5% during this study. We found a negative relationship between species diversity in one season and the proportion of deer mice that survived to the following season (e.g., Spring 2004 to Fall 2004; LMM, estimate = −0.147, Z = −2.738, *P*<0.01). There was a positive relationship between deer mouse persistence and SNV prevalence in deer mice (LMM, estimate = 0.44, Z = 2.40, *P* = 0.02).

### Potential Dilution or Amplification Agents

**Figure 1:**
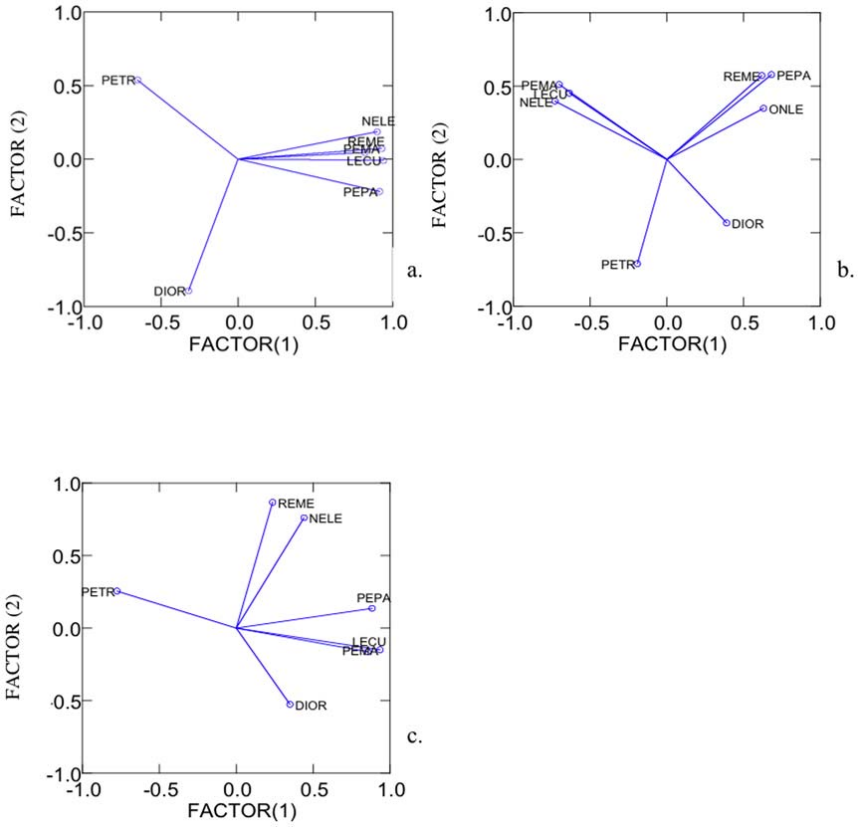
Principle components analysis (PCA) of rodent communities grouped by mean SNV prevalence. Total number of individuals of each species was entered as components. Factor loadings plots were standardized to two factors. Figure 1.a. Factor loadings plot for low (0–5%) prevalence communities. Figure 1.b. Factor loadings plot for moderate (7–14%) prevalence communities. Figure 1.c. Factor loadings plot for high (>17%) prevalence communities. PEMA = deer mouse, PETR = pinyon mouse, DIOR = Ord's kangaroo rat, NELE = desert woodrat, REME = western harvest mouse, PEPA = Great Basin pocket mouse, ONLE = Northern Grasshopper mouse.

Principle components analysis of the rodent communities indicated that pinyon mice and Ord's kangaroo rats have a significant influence on the dynamics of SNV prevalence in Great Basin rodent communities. For all prevalence categories, Factor 1 explained 35.8% to 65.6% of variance, whereas 16.8% to 29.1% was explained by Factor 2 (Fig. 1). Overall these factors explained between 62.0% and 82.4% of total model variance. For all prevalence categories, the total number of pinyon mice and total number of kangaroo rats appeared to have a strong influence on overall model variance, as these species segregated independently from all other species at all levels of prevalence.

There was a negative relationship between the number of pinyon mice per site and SNV prevalence (GLM, coefficient = −0.54, F_1, 39_ = 5.55, *P* = 0.02). In this model there was also a significant relationship between season and SNV prevalence on each site with prevalence generally increasing from spring to fall (GLM, F_5, 39_ = 2.78, *P* = 0.03). The relationship between the number of kangaroo rats per site and SNV prevalence was marginally significant (GLM, coefficient = 0.48, F_1, 39_ = 3.86, *P* = 0.06).

## Discussion

The goal of our study was to investigate the effect of species diversity on pathogen dynamics in nature. Our findings indicate that species diversity is negatively correlated with SNV prevalence in deer mice, following the predictions of the Dilution Effect hypothesis. This outcome could not have been predicted *a priori* in this system because other species can potentially host SNV and thus species diversity could have increased SNV prevalence. Deer mouse density and deer mouse persistence were lower at sites with higher species diversity, suggesting that these two mechanisms were important means through which species diversity may have shaped SNV prevalence. Below we discuss the mechanisms in greater detail, as well as the potential significance of particular species in the rodent community that may influence the dynamics of SNV.

Our findings corroborate other studies that implicate a negative relationship between species diversity and hantavirus prevalence. In a study of Choclo virus (a hantavirus found in Panama) [12], sites with reduced rodent species diversity (relative to a control) were found to have greater numbers of human cases of this strain of hantavirus, compared to other sites. Although they did not determine the prevalence of Choclo virus in the rodent community directly, their study underscores the importance of rodent community composition with respect to disease dynamics in humans. In Paraguay, antibodies for Laguna Negra virus (a hantavirus) were most prevalent in rodent communities with the greatest proportion of vesper mice (*Calomys laucha*), the primary reservoir [13]. Lastly, a longitudinal study of multiple SNV reservoir species in habitats across the southwestern US also reported a negative relationship between SNV prevalence and species diversity [38]. None of these studies addressed the mechanisms by which diversity may influence hantavirus prevalence.

### Species diversity, deer mouse density and SNV prevalence

Results of our study indicate that species diversity is negatively correlated with deer mouse density, but we did not find a significant relationship between deer mouse density and SNV prevalence. Other studies have also found that changes in rodent community composition had marked effects on deer mouse density [34], [36], [44]. Such changes in density may result from interspecific competition for nesting sites or food resources, from increased deer mouse dispersal away from sites, or increased predation risk as deer mouse movement and other behaviors change in the presence of other species [28]–[30].

### Species diversity, persistence of deer mice and SNV prevalence

Our study suggests that species diversity may influence SNV prevalence by reducing the persistence of deer mice at high diversity sites, as species diversity was negatively correlated with recapture probabilities of deer mice in subsequent seasons. Because this was a field study on free-ranging animals, we cannot definitively discriminate between two alternatives that could be driving this pattern, i.e. differential survival versus dispersal. However, further analysis of the data indicated that there was not a relationship between deer mouse body mass (a proxy for age) and species diversity, thus, differential survival seems less likely than differential dispersal (LMM, estimate = 0.88, Z = 1.09, P = 0.27). Fewer mice persisting at high diversity sites across seasons, indicates that these sites are less likely to have infected individuals remaining to maintain SNV in the population. We recognize that these conclusions are based on correlative studies and that further studies with the capacity for experimental manipulation are required to thoroughly test this hypothesis. However, these results are a necessary first step towards understanding the role of species diversity on SNV and identifying potential mechanisms.

Using a two-species mathematical model, Peixoto and Abramson [39] proposed that species diversity “dilutes” hantavirus prevalence through competitive pressure exerted by the non-host species on the host. The model differs from our findings, as their model implicates reduced host density as the mechanism by which species diversity modulates SNV prevalence whereas we found no direct connection with density. However, the possibility exists that competitive pressure from non-host species alters the retention (survival or dispersal) of deer mice on a site thereby impacting SNV prevalence.

Path analysis would provide further insight into the dynamics of the relationship between species diversity, deer mouse density and persistence, and SNV prevalence. However, path analysis is beyond the scope of the present study, as path analyses are more appropriate for larger data sets such as long-term longitudinal studies (e.g., >5 years, 25+ sites). Studies with larger and longer data sets may consider path analysis as a means to further understand the relationship between community ecology and the dynamics of pathogens in the wild.

LoGiudice and colleagues [9] suggested that pathogen prevalence could be influenced by a species other than the primary host, and coined this phenomenon a ‘dilution host’ A dilution host may be a species with low reservoir competency, transmitting the pathogen ineffectively and thereby reducing prevalence [9]. However, a dilution host, regardless of reservoir competency, may also reduce prevalence by altering the behavior of the primary host, such that contacts between hosts are infrequent and prevalence is therefore reduced [7]. Because these species can still affect prevalence without hosting the pathogen, we term them ‘dilution agents’, rather than hosts. Similarly, what we have termed an ‘amplification agent’ may be a species with high reservoir competency, transmitting the pathogen frequently and increasing overall prevalence [9]. However, an amplification agent may also be a species with low reservoir competency, yet the presence of this species may change the behavior of the primary host such that contacts between hosts are more frequent and prevalence subsequently increases [7].

We found that the number of pinyon mice in a community was negatively correlated with prevalence, indicating that they may serve as dilution agents. *A priori*, we predicted pinyon mice were likely amplification agents since they co-occur with deer mice in relatively high densities in the Great Basin and they are potential reservoirs for SNV (S. St. Jeor, unpublished). Measuring the competence of pinyon mice for SNV was beyond the scope of our study as it requires a Biosafety Level 4 facility. Perhaps pinyon mice serve as dilution agents for SNV by altering encounters between deer mice through changes in deer mouse movement, behavior or maturation. For example, Ovadia *et al*. [45] found a significant behavioral change within a species when that species was in the presence of another species. Male and female Allenby's gerbils (*Gerbillus andersoni allenbyi*) engaged in fewer aggressive interactions when in the presence of a larger-bodied competitor, *G. pyramidum*. Similarly, the presence of pinyon mice (a larger-bodied competitor) may decrease encounters between deer mice, and therefore reduce opportunities for SNV transmission. Alternatively, if pinyon mice are less competent reservoirs for SNV, then their encounters with deer mice may not result in successful transmission events, which would decrease prevalence, particularly if the total number of encounters regardless of species is fixed for deer mice. Lastly, if pinyon mice either directly or indirectly delayed the onset of reproduction in deer mice such that the population consisted of a greater proportion of younger individuals, SNV prevalence in deer mice could be reduced because juveniles are less likely to engage in behaviors that promote transmission [45], [46]. These hypotheses could be addressed in future field studies.

Our study also suggests that kangaroo rats, which do not host SNV, had a positive, though not statistically significant (*P* = 0.06), relationship with prevalence of SNV. Kangaroo rats may serve as amplification agents because they cause deer mice to have more interactions with one another. Falkenberg and Clarke [29] found that deer mice significantly shifted habitat use from open and closed microhabitats to almost exclusively closed microhabitats in the presence of kangaroo rats. The concentration of deer mouse activity within a microhabitat could increase SNV prevalence through the increased frequency of encounters.

The results of our study demonstrate the importance of community ecology in the dynamics of SNV prevalence in deer mice. In particular, our findings indicate that species diversity is negatively correlated with SNV prevalence and this relationship may be mediated by changes in deer mouse persistence and indirectly by deer mouse density. Our findings highlight the importance of understanding the behavior and ecology of disease reservoirs in the wild, to both predict future outbreaks and minimize the risk for human infection. We acknowledge the limitations of this study in that it was an observational study on free-ranging animals. However, it lays the necessary groundwork for future more manipulative studies that require substantial investment in enclosure infrastructure to execute experiments with replication at a landscape scale to address issues at the level of rodent communities. Furthermore, for pathogens such as SNV, that require BioSafety Level 4 laboratory facilities for experiments with infected animals, field studies can quickly advance our understanding of host-pathogen systems because the degree of safety precautions required in the laboratory are not needed for field studies.

### The Holy Grail: Contact Rates

For directly transmitted pathogens such as SNV, contact rates between infected and susceptible individuals represent the underlying mechanism through which diversity is predicted to act. Both the Dilution and Amplification hypotheses predict that species diversity will change the rate of contact between individuals. For many animals such as small, nocturnal animals contact rates are not easily obtained. While radiotelemetry may seem an obvious technology, the weight of the transmitters and the presence of observers can alter the behavior of rodents. Additionally telemetry location estimates are usually too large (within meters) to define probable contacts between small animals like rodents and the costs prohibit marking an entire rodent community. However, new technologies such as radio-frequency identification (RFID) devices such as passive integrative transponders coupled with inexpensive surveillance systems will make it possible in the future to document actual contact rates of such difficult to observe animals. These data will be extremely useful for the further assessment of the Dilution and Amplification hypotheses.

## Materials and Methods

### Study sites and sampling

Deer mice were sampled from 16 sites near the West Tintic Mountains in the Great Basin Desert of central Utah (Juab and Utah Counties) on public lands. To maintain independence, all sites were located >700 m apart. Study sites were dominated by big sagebrush (*Artemisia tridentata*) and Utah juniper (*Juniperus osteosperma*). We selected sites that varied in habitat heterogeneity based on shrub cover and bare ground, as both have been linked to diversity in rodent community composition [47]–[52]. Sites ranged from 1.2% shrub cover and 62.2% bare ground to 48.1% shrub cover and 6.1% bare ground. More site details can be found in Lehmer *et al*. [53].

Rodent sampling occurred in “Spring” (May and June) and “Fall” (late August and September, October) of 2003, 2004 and 2005, during 15 consecutive day periods that coincided with the new moon. Each site was trapped for three consecutive nights per season (Spring versus Fall). Although we monitored a total of 16 sites during our study, only 12 sites could be sampled during any single season due to time limitations. Of the 16 sites, five sites were sampled in every season and the remaining 12 sites were sampled in some, but not all seasons.

### Rodent Sampling

At each site, animals were live-trapped using 148 traps (H.B. Sherman Traps, Inc.) distributed in a “web” configuration over 3.1 hectares, following the methods of Mills *et al*. [54]. Upon capture, animals were identified to species; sex and weight were recorded. All animals were marked with uniquely numbered ear tags. Approximately 0.2 ml of blood was collected via the retro-orbital sinus of *P. maniculatus* at the time of initial capture for each trapping season. Blood was stored immediately on dry ice until transfer to an −80°C freezer. After blood collection, all animals were released at the point of capture. Some animals were captured multiple times during a single season (e.g., two of the three nights in spring), however only the data from the initial capture per season was used in the analysis. All workers implemented the recommended precautions for handling animals potentially infected with hantavirus [27] and all techniques for capturing and handling animals were approved by the Institutional Animal Care and Use Committee of the University of Utah (IACUC #0203011, #0503011).

### Species diversity, density and persistence estimates

Species diversity was estimated using Gini-Simpson Index (*D* = 1-∑ p^2^_i_; [53]). Density of deer mice at each site were estimated using the program DISTANCE (version 4.1) a software program designed to better estimate animal densities on web trapping designs than using the raw values [56]. We estimated persistence of deer mice as the percentage of individuals known to be alive from one trapping season to subsequent seasons on each site [40]; we were unable to estimate survival using programs such as MARK because it is often unreliable when recapture frequency is very low, as in the case of our study (often <10%).

### SNV antibody detection and estimates of prevalence

Enzyme linked immunosorbent assays (ELISA) were used to screen deer mouse blood for IgG antibodies to SNV. Because SNV is a chronic infection in deer mice, antibodies are a reliable indicator of SNV infection [57]. Briefly, wells of polyvinyl chloride microtiter plates (Dynatech Laboratories) were coated overnight at 4°C with recombinant nucleocapsid antigen diluted 1∶2000 in phosphate buffered saline. A non-hantavirus recombinant antigen was used as a negative control. After incubation, unbound antigen was removed from wells by washing 3× with wash buffer. Deer mouse sera were heat inactivated in a 55°C water bath for 30 minutes. Heat inactivated sera were diluted 1∶100 in serum-dilution buffer, containing powdered non-fat milk, Tween 20 and 10× PBS in a 1∶1∶20 ratio. The diluted serum solution was added to the antigen-coated wells and plates were then incubated at 37°C for 60 minutes. Plates were then washed 3 times with wash buffer (1∶20 Tween and 10× PBS) and incubated at 37°C for 30 minutes with 100 ul of ABTS Microwell Peroxidase Substrate Solution (Kirkegaard and Perry Laboratories, Inc.) [58]. Absorbance (405 nm) was recorded with a Versa Max Tunable Microplate Reader (VWR International) and values >3 standard deviations of the negative control wells on each plate were considered positive for anti - SNV antibodies [58]. Prevalence was estimated by dividing the number of unique seropositive deer mice by the total number of deer mice captured within a sampling period and this quotient was multiplied by 100. To eliminate the problem of overestimating prevalence through the inclusion of uninfected juveniles carrying maternal antibodies, prevalence was only calculated on the adults (>14 g) in the population [53].

### Statistical analysis

We first examined the relationship between SNV prevalence and species diversity using linear mixed models (LMM). LMM do not require balanced sampling of longitudinal repeated data (SYSTAT, Version 10, 2000; [59]), and thus was appropriate considering our unbalanced study design (e.g., sites repeatedly sampled, but not equally). In this analysis, species diversity was treated as a fixed effect, whereas site was considered a random effect to account for repeated sampling. Season could not be included as a factor in the analyses due to sample size (N = 16) limitations.

We next investigated the two potential mechanisms by which species diversity may influence SNV prevalence, also using LMM. In independent analyses, we evaluated the impact of species diversity on deer mouse density, as well as the effect of species diversity on the persistence of deer mice. In both analyses, species diversity was considered a fixed effect and site a random effect.

Finally, we examined whether these two mechanisms, deer mouse density and persistence were directly correlated with SNV prevalence. We evaluated the response of SNV prevalence to deer mouse density (fixed effect) with site as a random effect, using LMM. In a separate analysis, we evaluated the response of SNV prevalence to host persistence (fixed effect) with site as a random effect. We could not simultaneously evaluate the response of SNV to these two variables due to sample size limitations.

We used principle components analysis (PCA) to isolate key rodent species in our study system that had potential to influence SNV prevalence. In this analysis, we used the untransformed number of individuals of each species as components, since density estimates calculated by the program DISTANCE are not accurate for small samples sizes. Sites were grouped by categories according to mean prevalence across all sampling seasons (low (0–5%), moderate (7–14%), high (>17%)). We specified two factors for each analysis to standardize the factor loadings plots.

Based on the patterns identified by PCA, we used general linear models (GLM) to investigate the relationship between SNV prevalence and key rodent species. The total number of pinyon mice, kangaroo rats, and deer mice captured at each site were treated as independent continuous variables and site and season were independent categorical variables. Backward stepwise elimination was used to reduce general models to the most parsimonious version.
